# Anthracene phytotoxicity in the freshwater flagellate alga *Euglena agilis* Carter

**DOI:** 10.1038/s41598-019-51451-y

**Published:** 2019-10-25

**Authors:** Sreejith Kottuparambil, Jihae Park

**Affiliations:** 1 Red Sea Research Center, Division of Biological and Environmental Science and Engineering, King Abdullah University of Science and Technology (KAUST), Thuwal, 23955-6900 Saudi Arabia; 2 Ghent University Global Campus, Songomunhwa-Ro, 119, Yeonsu-gu, Incheon, 21985 Republic of Korea

**Keywords:** Environmental impact, Photosystem II

## Abstract

The freshwater flagellate alga *Euglena agilis* Carter was exposed to the polycyclic aromatic hydrocarbon (PAH) anthracene for 96 h under optimal photosynthetically active radiation (PAR), and responses of growth, photosynthetic pigment production, and photosynthetic efficiency were assessed. Anthracene reduced the growth rate (μ) and levels of chlorophyll *a* (Chl *a*), chlorophyll *b* (Chl *b*), and total carotenoids. The growth rate was more sensitive than photosynthetic parameters, with a median effective concentration (EC_50_) of 4.28 mg L^−1^. Between 5 and 15 mg L^−1^, anthracene inhibited the maximum quantum yield (*F*_v_/*F*_m_) of photosystem II (PSII) and the maximum photosynthetic electron transport rate through PSII (rETR_max_) with EC_50_ values of 14.88 and 11.8 mg L^−1^, respectively. At all anthracene concentrations, intracellular reactive oxygen species (ROS) were elevated, indicating increased oxidative stress. Anthracene presumably reduced the PSII efficiency of photochemical energy regulation and altered the photochemistry through intracellular ROS formation. Acute exposure to PAHs may induce severe physiological changes in phytoplankton cells, which may influence vital ecological processes within the aquatic environments. Additionally, growth and Chl *a* content may serve as sensitive risk assessment parameters of anthracene toxicity in water management since EC_50_ values for both overlap with anthracene levels (8.3 mg L^−1^) permitted by the US Environmental Protection Agency (USEPA).

## Introduction

Polycyclic aromatic hydrocarbons (PAHs) comprise a diverse family of hydrocarbons, each composed of two or more fused benzene rings. They are ubiquitous in terrestrial and aquatic ecosystems and are introduced by natural and man-made processes such as volcanic eruptions, crude oil spills, fossil fuel combustion, oil refining, automobile exhausts and industrial effluents^1^. PAHs are hydrophobic in nature and their persistence in the environment is a consequence of their low water solubility^2^. Due to their toxic, carcinogenic, mutagenic and photosensitising effects, especially their ability to generate singlet oxygen and other ROS, PAHs are considered one of the most prevalent groups of aquatic contaminants of high global concern^3^.

Anthracene is a three-ring, low molecular weight PAH with relatively high water solubility than other toxic PAHs^4^. Anthracene adversely affects the growth and photosynthesis of natural phytoplankton communities, with a toxicity threshold value less than its aqueous solubility^1^. Moreover, it is one of the most rapidly modified hydrocarbons and is assumed to be a strong photosensitizer^5^ that induces intracellular oxidative stress and blockage of the photosynthetic electron transport chain^6^ through the formation of ROS. Given the wide occurrence and high toxicity to aquatic organisms, anthracene is now on the list of priority pollutants, with a recommended water quality criterion of 8.3 mg L^−1^ and interim water quality guidelines of 0.012 μg L^−1^ for the protection of freshwater life set by the Canadian Council of Ministers of the Environment^7,8^.

Microalgae are an important group of primary producers in aquatic habitats that play a pivotal role in aquatic ecosystems, forming the food and energy base for all organisms, and powering food webs and biogeochemical cycling. They are readily exposed to toxic waterborne contaminants and tend to reach an equilibrium with pollutants rather rapidly because they are small with a proportionally large surface area^9^. Various studies have reported the toxic effects of PAHs to freshwater algae in terms of growth, photosynthesis, and respiration, with special attention given to interactions with solar and ultraviolet (UV) radiation^10–12^. However, Brack *et al*.^13^ suggested that anthracene toxicity is largely independent of irradiation and the intact compound itself contributes to toxicity. EC_50_ values reported for anthracene in freshwater microalgae range between 0.024 and 5 mg L^−1^ (Table 1). Among the various toxicity criteria studied, inhibition of photosynthesis is particularly pertinent as it inevitably results in reduced growth, biomass yield and loss of competitive ecological advantage.

**Table 1:** Anthracene toxicity data for freshwater microalgae.

Tested taxa	Criterion	Test period	Effect (mg L^−1^)	Reference
*Chlamydomonas angulosa*	Photosynthesis	3 h	0.54, EC_50_	^42^
*Chlorella vulgaris*	Photosynthesis	3 h	0.24, EC_50_	
*Selenastrum capricornutum*	Growth	96 h	>40, EC_50_	^63^
*Pseudokirchneriella subcapitata*	Growth	34 h	0.037, EC_50_	^1^^ *^
*P. subcapitata*	Primary production	36 h	0.024, EC_50_	
*Chlorella protothecoides*	Growth	96 h	0.85, EC_50_	^64^
Natural Phytoplankton	*F*_v_/*F*_m_	30 min	<0.2, EC_50_	^10^
*Chlorella vulgaris*	Growth	96 h	1.27, EC_50_	^10^
*Coenochloris pyrenoidosa*	Growth	96 h	1.47, EC_50_	^10^
*Scenedesmus subspicatus*	Growth, area under curve	7 d	1.04, EC_50_	^65^
*S. armatus*	Population density	24 h	0.25, EC_50_	^32^
*S. vacuolatus*	Population density	24 h	0.5, EC_50_	^11^
*Scenedesmus spp*.	Growth	24 h	0.25, significant reduction	^66^
*Chlamydomonas reinhardtii*	Population density	24 h	0.28, EC_50_	^38^
*Desmodesmus subspicatus*	Growth	72 h	0.26, EC_50_	^67^
*Microcystis aeruginosa*	Growth	72 h	>0.06, significant reduction	^68^
*Anabaena fertilissima*	Growth	8 d	5.0, EC_50_	^62^

^*^Under UV-A radiation (12.5 Wm^−2^).

Currently, most countries have legislation and regulations on accepted values for toxicity derived from bioassays that are applied to regulate agricultural and industrial chemicals, biocides, cosmetics, food additives, medicines and other substances^14^.

The genus *Euglena* contains motile, unicellular, photosynthetic eukaryotes found in many aquatic habitats, especially shallow eutrophic water bodies. These flagellates have rapid growth rates and can be easily cultured in the laboratory at low cost, ensuring year-round availability. *Euglena* spp. are sensitive to physicochemical changes and pollution in the surrounding environment, therefore, are potentially used as model organisms in ecotoxicological studies^15,16^. Widely used endpoints in bioassays involving *Euglena* are, growth inhibition^17^, photosynthesis and respiration^18^, chlorophyll content^19^, gene expression^20^ and motility/orientation^21^. Despite large quantities of toxicity data from the analysis of pollutants such as metals and herbicides^21–23^, the effects of PAHs on *Euglena* remain poorly understood.

The ultimate goal of bioassay tests is to provide representative and incorporative criteria regarding exposure conditions, thereby improving risk assessment and management of water quality. In this respect, multiple, rather than single, endpoint assays may be more reliable for comprehensive risk assessment of toxicants. Such an approach may facilitate insight into the mechanisms of toxicity and provide information on the relative sensitivity of selected parameters to toxicant concentration and/or exposure duration, thereby establishing methods for detecting changes caused by particular phytotoxicants^24^. In the present study, we investigated the ecotoxicological effects of anthracene on three endpoints of *Euglena agilis* Carter, including growth, pigmentation, and Chl *a* fluorescence which were then compared with permitted levels of anthracene in aquatic environments set by the US Environmental Protection Agency (USEPA). Phytoplankton is the main biomass producers in aquatic ecosystems, contributing ca. 50% of the atmospheric carbon dioxide fixation^25^. Any negative effects of anthracene on the growth and photosynthesis of phytoplankton would be detrimental to entire aquatic ecosystems and food chains.

## Materials and Methods

### Algal test species and culture conditions

*Euglena agilis* Carter was cultured in mineral medium (pH 5)^26^ in 1 L Erlenmeyer flasks at 25 °C under white fluorescent irradiance (PAR; 400–700 nm) of 30 µmol photons m^–2^ s^−1^ (FL400, Kum-Ho, Seoul, Korea) on a 16:8 h light:dark (LD) cycle. All experiments were performed using cells at the exponential growth phase.

### Test chemicals and exposure

Anthracene (99% purity, CAS No. 120–12–7) was purchased from Sigma Aldrich (Saint Louis, MO, USA) and test solutions at the desired concentrations were prepared by serial dilution from stocks in high-performance liquid chromatography (HPLC)-grade dimethyl sulphoxide (DMSO; Sigma Aldrich). Microplate toxicity tests of 96 h in duration were conducted in 24-well cell culture plates (well diameter = 15.6 mm, growth area = 1.9 cm^2^; SPL Life Sciences, Gyeonggi-Do, Korea) with a test volume of 2 mL per well. Equal volumes of cell suspension and anthracene stock solutions were mixed to obtain final concentrations of 0.625, 1.25, 2.5, 5, 10 and 15 mg L^−1^, along with untreated controls. The concentration of the carrier solvent did not exceed 0.2% v/v of the test culture volume. The initial cell density was 10 ± 0.5 × 10^4^ cells mL^−1^ of suspension. An additional solvent toxicity test (96 h) was conducted with a maximum DMSO concentration of 0.2% v/v. Organisms were exposed to nominal concentrations of anthracene and all treatments were performed in triplicate. The well plates were covered with parafilm to avoid evaporation and mixing of the volatile toxicant.

### Measurement of growth rate

Growth rates were determined by measuring the number of cells in each well on the first and final days using a hemocytometer (Marienfeld, Germany). The specific growth rate (μ) was calculated using the following formula:

$${\rm{\mu }}=\frac{\mathrm{LN}(\frac{N2}{N1})}{(t2-t1)}$$

where *N*_1_ and *N*_2_ are the number of cells at time *t*_1_ (initial) and *t*_2_ (final), respectively.

### Estimation of photosynthetic pigments

Photosynthetic pigment content was estimated using standard protocols^27^. Briefly, 1 mL cell suspension was collected from each replicate culture and centrifuged before extraction, and 1 mL of 90% v/v acetone was added followed by vigorous vortexing and centrifugation at 10,000 × g for 5 min at 4 °C. Supernatants were withdrawn and their optical density was measured spectrophotometrically at 470, 664 and 647 nm using an S-3100 UV/Vis spectrophotometer (Scinco, Seoul, Korea). Pigment concentrations are expressed as μg mL^−1^ of suspension.

### Measurement of chlorophyll a (Chl a) fluorescence

Chl *a* fluorescence was measured using a pulse amplitude modulation (PAM) imaging instrument (Walz, Germany) as a proxy for photosynthetic performance. For measurement of maximum quantum yield (*F*_v_/*F*_m_) and electron transport rate (ETR), samples were kept in the dark for 10–15 min and then subjected to pulsed light emitted by a diode at ~0.15 µmol photons m^−2^ s^−1^ to obtain the initial fluorescence yield (*F*_o_), which denotes the fluorescence yield when all PSII reaction centres are open with fully oxidized plastoquinone A(QA). A saturation pulse of ~5000 µmol photons m^−2^ s^−1^ emitted by a built-in halogen lamp was then applied to produce the maximum fluorescence yield (*F*_m_), which is induced by a short saturating pulse of actinic light that reduces all QA. The maximum PSII quantum yield (*F*_v_/*F*_m_) was then derived from the equation (*F*_m_ − *F*_o_)/*F*_m_.

Rapid light curves were produced using 10 s pulses of actinic light increased stepwise from 0 to 335 (0, 1, 11, 21, 36, 56, 81, 111, 146, 186, 231, 281 and 335) μmol photons m^−2^ s^−1^. The relative electron transport rate (rETR) was calculated by multiplying the effective quantum yield (ΦPSII = *F*′_m_ − [*F*/*F*′_m_], where F′_m_ is the maximum light-acclimated fluorescence yield and F is the light-acclimated fluorescence yield) by photon flux density (PFD) and plotting against PFD. The ETR is relative because the absorbance of light by cells was not measured. Maximum electron transport rate (ETR_max_) was derived from the hyperbolic tangent formula rETR = ETR_max_ ∗ tanh (α/I/ETR_max_), adapted from Jassby and Platt^28^, where α indicates the electron transport rate under light-limited conditions. Alterations in Chl *a* fluorescence due to changes in non-photochemical quenching (NPQ) and photochemical quenching (qP) were calculated from (*F*_m_ − *F*′_m_)/*F*′_m_ and (*F*′_m_ − *F*_t_)/(*F*′_m_ − *F*′_o_), respectively^29^.

### Measurement of ROS levels

The oxidant-sensing fluorescent probe 2′,7′-dichlorodihydrofluorescein diacetate (DCFH-DA; Sigma Aldrich; CAS No: 4091–99–0) was used to detect intracellular ROS generation in *E. agilis* treated with anthracene. DCFH-DA (5 μM, final concentration) solubilized in ethanol was added to the cell suspension and incubated on a shaker at room temperature in the dark for 1 h^30^. The fluorescence intensity was measured at an excitation wavelength of 485 nm and an emission wavelength of 530 nm using a Spectra MAX Gemini EM microplate fluorescence reader (Molecular Devices, CA, USA). The relative production of ROS is represented as relative fluorescence units (RFU).

### Statistical analyses

Data are presented as means ± 95% confidence intervals (CI). All parameters were compared across treatments with one-way analysis of variance (ANOVA, n = 3, *p* < 0.05) using the JMP software (JMP^®^ Pro version 13.1, SAS Institute, USA). Multiple comparison tests based on the least significant difference (LSD) were then carried out to find significant differences (*p* < 0.05) from controls and between treatments. The effective concentration at which 50% inhibition occurs (EC_50_) was estimated by the linear interpolation method using ToxCalc 5.0 (Tidepool Science, USA). The coefficient of variation (CV), the standard deviation expressed as a percentage of the mean, was calculated to estimate the precision of test values.

### Capsule

Anthracene significantly reduces growth and photosynthesis in the freshwater flagellate Euglena agilis via intracellular ROS generation.

## Results and Discussion

### Effect of anthracene on cell growth

**Figure 1:**
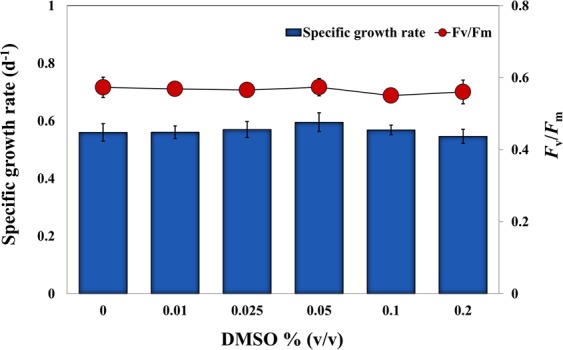
Effect of the carrier solvent DMSO on growth and photosynthesis (*F*_v_/*F*_m_) of *E. agilis*. A maximum concentration of 2% (v/v) was tested.

The carrier solvent used in this study (DMSO) had no significant inhibitory effects on cell growth (ANOVA, *df* = 6, *F* = 1.3, *P* > 0.05) or photosynthetic efficiency (ANOVA, *df* = 5, *F* = 0.56, *P* > 0.05) of *E. agilis*, even at the maximum concentration of 0.2% (v/v) in the growth medium (Fig. 1). Okumura *et al*.^31^ previously demonstrated the suitability of DMSO as a carrier solvent in Euglenoid tests.

**Figure 2:**
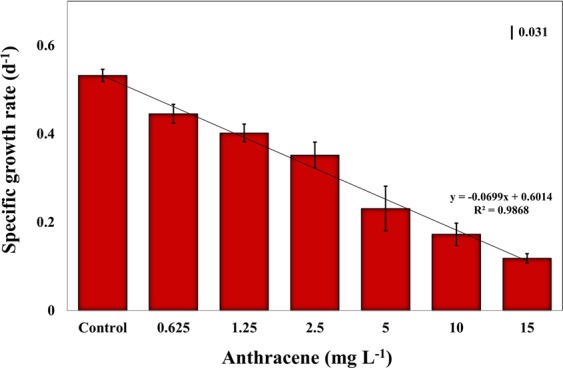
Effect of 96 h anthracene exposure on the specific growth rate (days^−1^) of *E. agilis*. Mean and 95% confidence intervals (CI) are shown (n = 3). Vertical bar indicates LSD, least significant difference.

Most of the toxicity data available for the effects of anthracene on freshwater microalgae are based on growth inhibition (Table 1). Growth is an important endpoint parameter that reflects the overall vitality of a population under the tested conditions. Addition of anthracene to the culture medium resulted in a concentration-dependent inhibition of the specific growth rate of *E. agilis* (Fig. 2). Compared with controls, the final day cell densities were significantly lower at all tested concentrations, and μ was significantly reduced from 0.53 for control cells to 0.12 at the highest anthracene dose (ANOVA, *df* = 6, *F* = 198.82, *P* < 0.001). The EC_50_ value for growth was 4.28 mg L^−1^ (Table 2), which is greater than the values previously reported for several freshwater microalgae (Table 1). At nominal concentrations exceeding 0.05 mg L^−1^, anthracene significantly inhibits the growth of freshwater phytoplankton^32^. For example, the growth of *Selenastrum capricornutum* was extremely sensitive to anthracene, aggravated by UV radiation, with a 22 h EC_50_ value of 3.9–37.4 μg L^−1 ^^33^. Our results suggest that anthracene itself is potentially toxic to freshwater primary producers, and could add synergistic effects with other stressors such as UV radiation^32^.

**Table 2:** NOEC, LOEC and EC values (mg L^−1^) plus CI and *p*-values for inhibition of *E. agilis* exposed to anthracene for 96 h.

Test Criterion	NOEC	LOEC	Mean EC_10_, 95% CI and CV (%)	Mean EC_50_, 95% CI and CV (%)	*p-*value
Growth rate (μ)	<0.625	0.625	0.38 (0.3–0.55) 10.10	4.28 (3.58–5.14) 6.63	<0.05
Chl *a*	<0.625	0.625	0.31 (0.12–0.22) 8.46	5.59 (2.84–7.98) 11.77	<0.05
Chl *b*	<0.625	0.625	0.34 (0.16–0.63) 16.56	8.14 (5.52–10.73) 7.99	<0.05
Carotenoids	<0.625	0.625	0.41 (0.34–0.54) 6.35	>15	<0.05
*F*_v_/*F*_m_	2.5	5	3.18 (0.93–4.93) 14.88	13.74 (11.90–15.65) 3.41	<0.05
rETR_max_	2.5	5	2.16 (0.89–5.52) 15.57	11.80 (8.92–14.68) 7.43	<0.05

^*^NOEC, no observed effect concentration; LOEC, lowest observed effect concentration; CI, confidence interval; CV, coefficient of variation.

^*^Mean and 95% CI are shown (n = 3).

Growth inhibition due to PAH exposure in microalgae and higher aquatic plants has been previously reported^34^, and the extent of growth inhibition depends on the species studied, the chemicals tested and the duration of exposure. Reduction in growth can result from an accumulation of anthracene within the lipid fraction of cells and subsequent changes in membrane properties^35^. PAH accumulation in membranes can cause an expansion of the membrane surface area, inhibition of primary ion pumps, and an increase in proton permeability, leading to dissipation of the electrical potential and pH gradient, which ultimately results in inhibition of cellular growth^36^. Additionally, a reduction in photosynthesis can lead to impaired growth, since these are highly interrelated phenomena, each being a function of the utilization of energy from light and nutrients. Even moderate changes in the function of the photosynthetic apparatus can lead to a marked reduction in energy production within chloroplasts^34^.

### Effect of anthracene on pigment content

**Figure 3:**
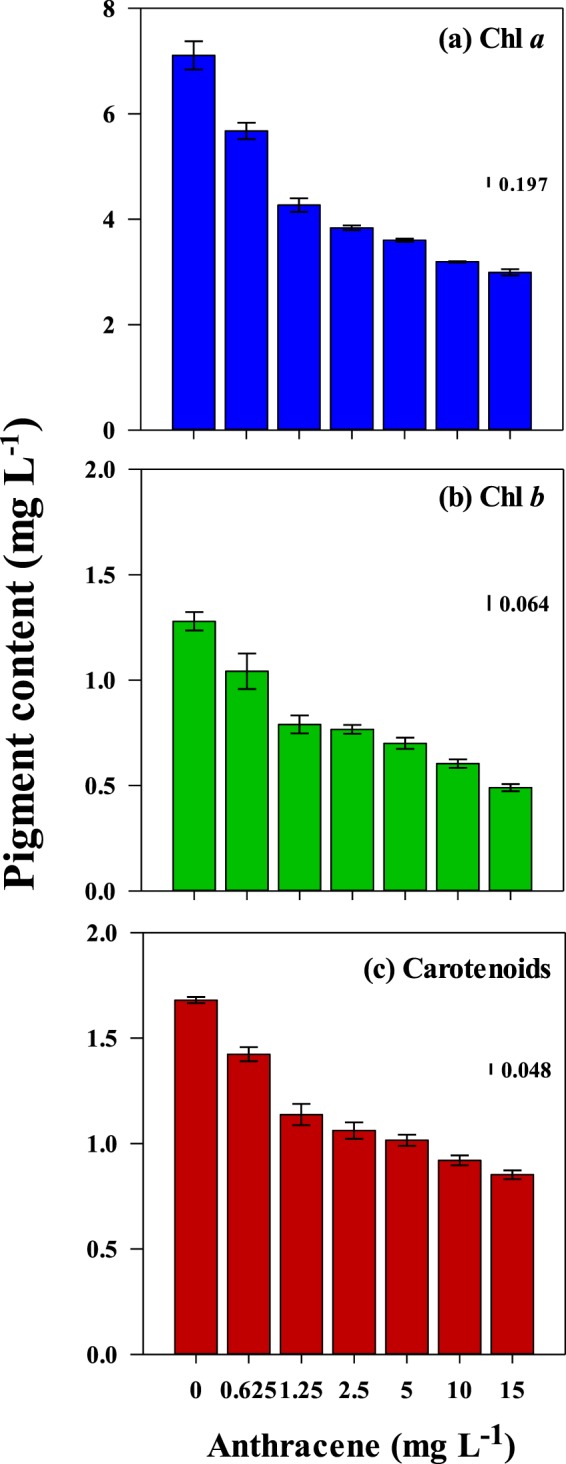
Effect of exposure to anthracene for 96 h on photosynthetic pigmentscontent in *E. agilis;* (**a**) Chl *a*, (**b**) Chl *b*, and (**c**) total carotenoids. Mean and 95% confidence intervals (CI) are shown (n = 3). Vertical bars indicate LSD, least significant difference.

*Euglena* contains both Chl *a* and *b* as light-harvesting pigments, along with the carotenoids, diadinoxanthin, and diatoxanthin^37^. Despite studies on the effect of anthracene on growth and photosynthesis in algae, limited information is available on their interference with photosynthetic pigment production. Anthracene did not affect chlorophyll biosynthesis in *Chlamydomonas reinhardtii* strain cw92 at concentrations up to 1 mg L^−1 ^^38^, or in three *Desmodesmus* spp. up to 0.25 mg L^−1 ^^37^. However, in the present study, anthracene (>0.625 mg L^−1^) had a pronounced effect on photosynthetic pigments content in *E. agilis*. The most abundant pigment in *E. agilis* was Chl *a* (7.14 μg mL^−1^) followed by carotenoids (1.72 μg mL^−1^) and Chl *b* (1.25 μg mL^−1^). At the lowest test concentration (0.625 mg L^−1^), there were significant reductions in Chl *a*, Chl *b* and total carotenoids of up to 20%, 16%, and 17%, respectively, while at the highest concentration, reductions of 58%, 64%, and 49% were observed (Fig. 3). The adverse effect on pigment content was concentration-dependent, with 96 h EC_50_ values of 5.59 mg L^−1^, 8.14 mg L^−1^ and >15 mg L^−1^ for Chl *a* (ANOVA, *df* = 6, *F* = 334.54, *P* < 0.05), Chl *b* (ANOVA, *df* = 6, *F* = 40.05, *P* < 0.05) and total carotenoids (ANOVA, *df* = 6, *F* = 130.11, *P* < 0.05), respectively (Table 2).

The molecular mechanism of the reduction in pigment levels may involve the accumulation of lipophilic anthracene in thylakoid membranes^39^, resulting in conformational changes in their structure and composition. In general, reduced pigmentation under chemical stress results from inhibition of enzymes related to chlorophyll synthesis, degradation of chlorophyll and DNA damage^40^, or accelerated degradation of pigments due to increased ROS formation at various positions in the photosynthetic electron transport chain. Moreover, carotenoids prevent photo-oxidative destruction of chlorophylls^41^ and, therefore, a reduction in carotenoids could have additional serious consequences on chlorophyll molecules. The simultaneous reduction in all three photosynthetic pigments suggests that the major target of anthracene toxicity is the thylakoid compartment of chloroplasts. These results also indicate that in addition to causing a severe reduction in growth, anthracene exposure may reduce photosynthetic performance via the destruction of pigments responsible for harvesting available photons.

### Inhibition of photosynthesis

**Figure 4:**
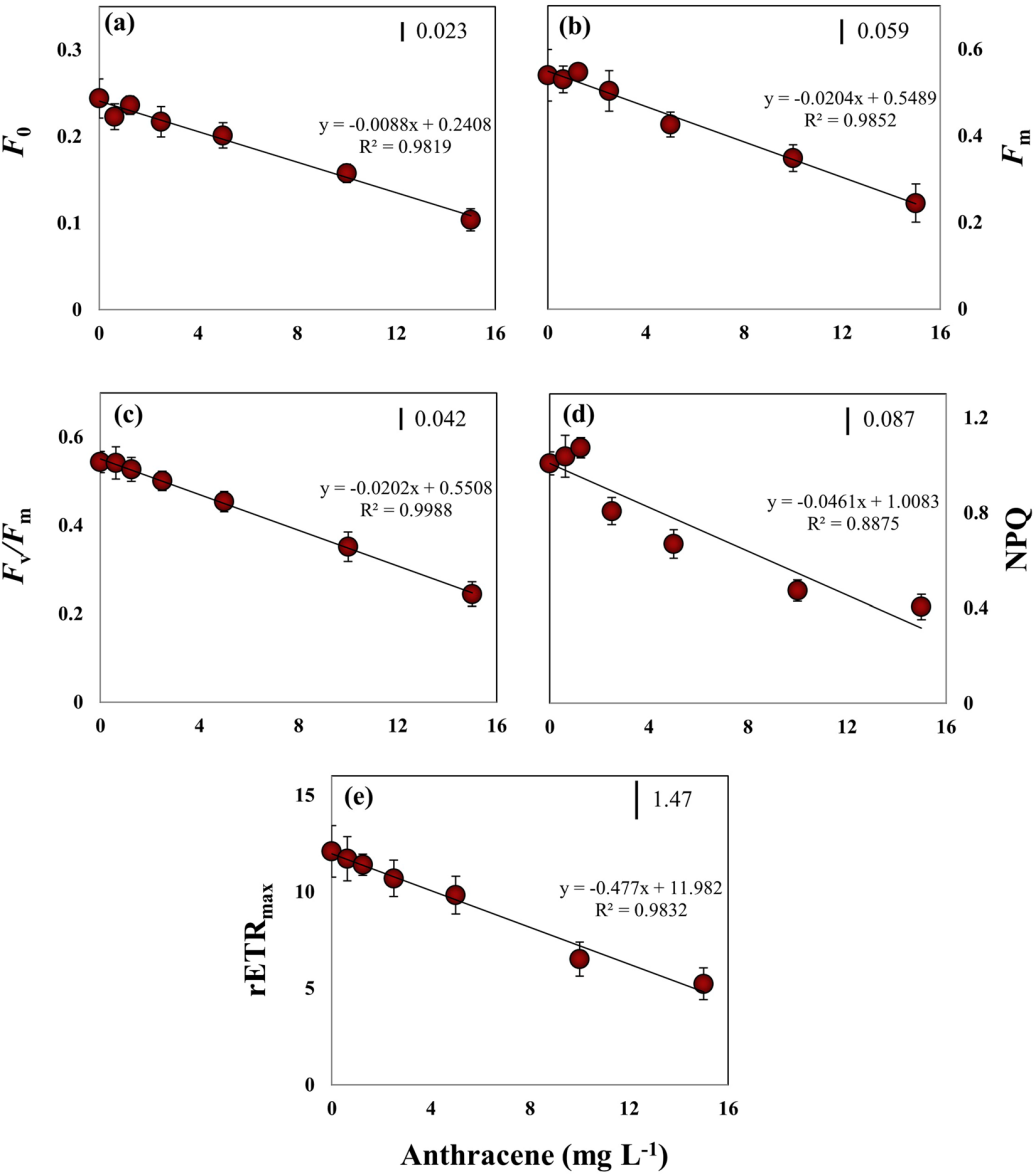
Effect of exposure to anthracene for 96 h on Chl *a* fluorescence parameters of *E. agilis*. (**a**) Minimum fluorescence (*F*_o_). **(b**) Maximum fluorescence (*F*_m_). (**c**) Maximum quantum yield of PSII (*F*_v_/*F*_m_), (**d**) Non-photochemical quenching (NPQ), (**e**) Maximum relative electron transport rate (rETR_max_). Mean and 95% confidence intervals (CI) are shown (n = 3). Vertical bars indicate LSD, least significant difference.

Anthracene is a strong inhibitor of phytoplankton photosynthesis *in vivo*^32,35,38,42^. We conducted *in vivo* Chl *a* fluorescence measurements as an intriguing tool to reveal the toxic effects of anthracene on the photosynthetic machinery of *E. agilis* (Fig. 4). The quantum yield and quantum efficiency parameters are indicators of the efficiency of solar energy absorption, which decreases under chemical stress, implying that stress negatively impacts photon absorption and conversion of solar energy during photosynthesis^43^. We found that at higher anthracene concentrations (>5 mg L^−1^), there were significant reductions in dark fluorescence (*F*_0_; ANOVA, *df* = 6, *F* = 40.90, *P* < 0.05), which reflects emission by excited Chl *a* molecules in the antennae structure of PSII, and in maximal fluorescence (*F*_m_; ANOVA, *df* = 6, *F* = 33.54, *P* < 0.05), which can be attributed to severe loss of pigments and/or inactivation of PSII reaction centres. It is evident that at higher anthracene doses, reduction in the number of cells and pigment levels resulted in an overall decline in the light-harvesting by *E. agilis*. Moreover, no significant variation (ANOVA, *df* = 3, *F* = 2.01, *P* > 0.05) in *F*_0_ was observed between 0–2.5 mg L^–1^ anthracene, despite significant reductions in the concentration of Chl *a* (Fig. 3a), suggesting that pigment molecules associated with PSII reaction centres are less affected. Instead, anthracene may pose a more serious threat to the pigment pool of PSI. This interpretation is supported by the findings of Huang *et al*.^44^, who suggested that PSI is the primary site of action of anthracene. However, Chl *a* fluorescence measurements in plants and algae have suggested inhibition of the cytochrome-b6/f complex and/or photo-oxidative damage to PSII as additional modes of anthracene toxicity^6,38^.

*F*_v_/*F*_m_, an estimate of the photochemical conversion efficiency of PSII in the dark, has been widely used to assess the acute toxicity of aromatic hydrocarbons in freshwater plants and phytoplankton^6,35^. An *F*_v_/*F*_m_ value of ~0.55 relative units (RU) was recorded in our control *E. agilis* population, comparable to the value reported previously for *Euglena gracilis*^21^. *F*_v_/*F*_m_ did not significantly vary up to 1.25 mg L^−1^ anthracene (Fig. 4c). However, at higher concentrations, *F*_v_/*F*_m_ was declined (ANOVA, *df* = 6, *F* = 63.18, *P* < 0.05) by 17% (5 mg L^−1^), 36% (10 mg L^−1^), and 55% (15 mg L^−1^) with an EC_50_ of 13.74 mg L^−1^ (Table 2). Toxicity of anthracene on *F*_v_/*F*_m_ in microalgae taxa has not been reported previously, so direct comparison of the sensitivity of *E. agilis* with other species is not possible. Nevertheless, in the macrophyte *Lemna gibba*, *F*_v_/*F*_m_ appeared to be a more sensitive biomarker of anthracene toxicity, with a 4 h EC_50_ value of 2 mg L^−1 ^^44^.

We noted that the reduction in *F*_v_/*F*_m_ at >2.5 mg L^−1^ anthracene was accompanied by a significant loss of NPQ (Fig. 4d). Although values were not statistically significant, NPQ tended to increase up to 1.25 mg L^−1^ and then decreased significantly thereafter (ANOVA, *df* = 3, *F* = 44.74, *P* < 0.05). This decline in Chl *a* fluorescence quenching can be attributed to impairment of electron transport downstream from PSII and an elevated reduction of the PQ pool^45^. NPQ is produced through the generation of an H^+^ electrochemical gradient across the thylakoid membranes^46^ and is an indicator of absorbed energy that is dissipated through heat loss and other non-photochemical mechanisms. We assume that a severe reduction in the photosynthetic process at high anthracene levels likely reduces the magnitude of the pH gradient, thereby affecting NPQ values, consistent with the view of González–Moreno *et al*.^47^ who reported similar observations on fluorescence quenching in *E. gracilis* under salt stress. Severe reduction in *F*_v_/*F*_m_ of *E. gracilis* exposed to NaCl had resulted in a diminution of the pH gradient across the thylakoid membranes and subsequently, up to 95% reduction in NPQ^47^.

In the present study, anthracene reduced the ETR across PSII at higher concentrations (Fig. 4e). rETR is an empirical estimate of the rate of the flow of electrons through the electron transport chain. rETR_max_ was not significantly affected up to 1.25 mg L^−1^ anthracene, whereas 5, 10 and 15 mg L^−1^ doses resulted in 18%, 46%, and 56% reductions, respectively, with an EC_50_ value of 11.8 mg L^−1^ (Table 2). Thus, the threshold value of anthracene for a significant reduction in ETR was higher than that for growth inhibition. In aquatic plants, PAHs inhibit photosynthetic electron transport at concentrations below which growth and CO_2_ fixation are inhibited^44^. For PAHs in general, the target of their toxicity to photosynthesis is the electron transport downstream from PSII, specifically at cytochrome-b6f. Inhibition of electron transport blocks reoxidation of the reduced plastoquinone pool (PQH_2_) and the absorbed energy cannot be used in photochemistry^6^. A probable consequence of inhibition of the electron transport chain at PSII is the transfer of energy from triplet chlorophylls to oxygen, forming singlet oxygen species, which induces oxidative damage of cells^48^. The generation of free radicals and subsequent intracellular oxidative stress is a prominent mechanism of anthracene toxicity in freshwater phytoplankton^38^.

**Figure 5:**
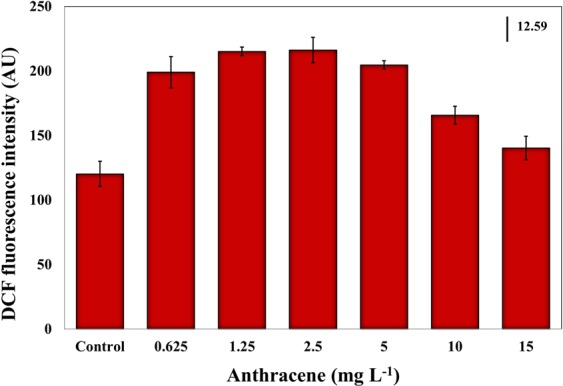
Effect of exposure to anthracene for 96 h on DCFH-DA fluorescence intensity. The excitation wavelength was 485 nm and the emission wavelength was 530 nm. Mean and 95% confidence intervals (CI) are shown (n = 3). Vertical bar indicates LSD, least significant difference.

When DCFH-DA fluorescence emission in anthracene exposed *E. agilis* cells was measured, a significant increase was observed at all dosages (ANOVA, *df* = 6, *F* = 81.11, *P* < 0.05), indicating a rise in intracellular ROS levels (Fig. 5). ROS level at 2.5 mg L^−1^ anthracene was almost double than that in the controls. The subsequent reduction in fluorescence at high anthracene (>5 mg L^−1^) can be attributed to reduced cell growth and diminished enzyme activities, although values were still significantly higher (*p* < 0.5) than in controls. The major site of ROS production in photosynthetic organisms is the disrupted electron transport chain across PSII^49^. We report here, for the first time in freshwater microalga taxa, the significant elevation of intracellular ROS levels under anthracene stress. In *Euglena* spp., ROS play a significant role in metal toxicity^45^, UV damage and defense mechanisms^50^. However, ROS, generated by chemical stressors, trigger adverse effects through multifaceted actions inside the cell. They attack thylakoid lipids and initiate peroxyl radical chain reactions, eventually destroying membranes and pigment-protein complexes^45^. Moreover, in chloroplasts, ROS cause lipid peroxidation, which results in the disruption of photosynthetic pigments, and the inactivation and degradation of RuBisCo and other components of the Calvin cycle^51^. Our results correspond to Babu *et al*.^52^, who found that 1,2-dihydroanthraquinone, a photoproduct of anthracene, inhibited photosynthetic electron transport, leading to the overproduction of O_2_^−^ and subsequent oxidation of proteins, membranes, and pigments in *Lemna gibba*.

DCFH-DA is more suitable to estimate total ROS production rather than as a probe for a particular type of ROS^53^. The superoxide anion radicals (O_2_^−^) produced in the electron transport chain is a precursor for many other ROS species. They are rapidly converted to hydrogen peroxide (H_2_O_2_) and subsequently to hydroxy radicals (OH^•^) by enzymatic reactions^54^. DCFH-DA does not directly react with O_2_^−^ but can be oxidized to highly fluorescent DCF by H_2_O_2_ and OH^•^ radicals^55^. Thus, DCFH-DA probing of ROS revealed the overall cellular redox status of *E. agilis* under anthracene stress. Higher ROS also reflect the inefficiency of both photochemical pathways and protective regulatory mechanisms to process the excitation energy at PSII. Our data suggest that ROS generation and consequential oxidative stress play a pivotal role in acute anthracene toxicity in the model organism, *E. agilis*. We detected significant ROS levels under optimal PAR irradiation, where photo-modification of the parent compound is less likely. Under high oxidative damage, *Euglena* relies on the activation of antioxidant enzymes such as ascorbate peroxidase (APX) and glutathione peroxidase (GPX)^56^, and biosynthesis of antioxidant metabolites such as reduced glutathione (GSH) and its derivatives^57^. Furthermore, some canonical metabolites act as indicators of oxidative damage, such as malondialdehyde (MDA)^58^. Thus, antioxidant/oxidant responses upon anthracene exposure may represent a promising area for further investigation.

**Figure 6:**
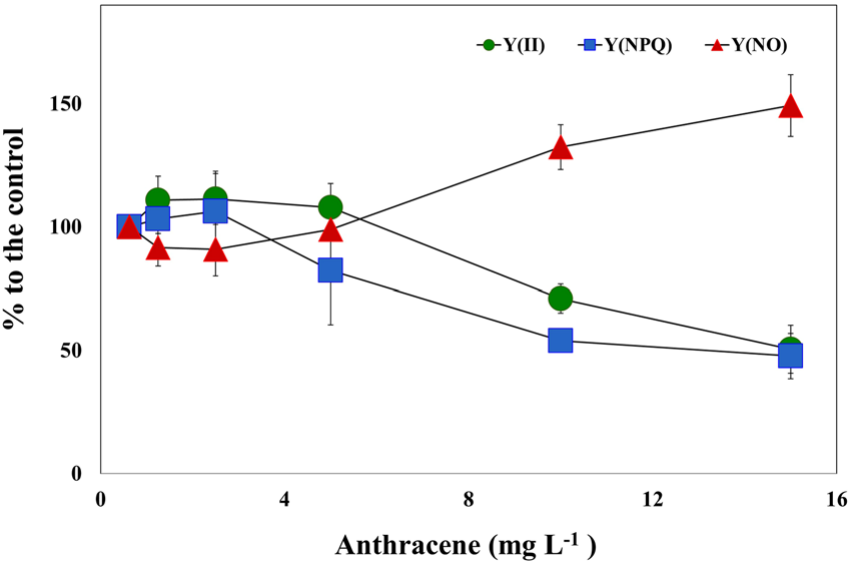
Effect of exposure to anthracene on overall energy conversion at PSII in terms of three quantum yields; (i) photochemical quantum yield of photosystem II, Y(II); (ii) quantum yield of non-photochemical fluorescence quenching due to downregulation of the light harvesting function, Y(NPQ); (iii) quantum yield of non-photochemical fluorescence quenching other than that caused by down-regulation of the light harvesting function, Y(NO). Values are given as % relative to untreated controls.

We further analyzed the three photochemical quantum yields of PSII measured by imaging PAM to describe the response of PSII photochemistry to anthracene (Fig. 6). Y(II) represents the fraction of excitation energy converted photochemically at PSII. The remaining fraction, 1–Y(II), is the sum of the yields of regulated dissipation, referred to as Y(NPQ), and unregulated dissipation, indicated by Y(NO)^59^. As stated before, anthracene reduced the *F*_v_/*F*_m_ in a dose-dependent manner, and the photon energy requirements for a complete reduction of Q_A_ decrease drastically. This explains the gradual reduction in Y(II) with increasing anthracene doses and a corresponding increase in Y(NO) (Fig. 6). Y(NO) denotes the excess energy, the fraction of absorbed energy used for the generation of free radicals (ROS) via an apparent catalytic transfer of electrons occurred from the reduced PQ pool to O_2_. The higher quantum yield of non-regulated non-photochemical energy loss of PSII (Y(NO)) is a significant stress response, suggesting potential damage to the photosynthetic apparatus exerted by anthracene. Moreover, the declining Y(NPQ) is an indicator of the failure in regulated non-photochemical quenching mechanisms to process the excess energy at PSII. These PSII quantum yield parameters collectively indicates the reduced efficiency of photochemical energy regulation imposed by anthracene exposure.

On the downside of our methodology, anthracene has a higher n-octanol/water partition coefficient (K_OW_) of 4.54^60^ and, therefore, multi-well plate assay is likely to underestimate the toxicity potential, because a loss of exposure concentration due to lipophilicity is expected for hydrophobic compounds with K_OW_ > 4^61^. We controlled the evaporative loss of the toxicant by sealing the well plates, however, loss in nominal concentration due to physicochemical properties of the tested PAH may have adversely affected the toxicity thresholds reported here. Nevertheless, our data confirmed the mode of anthracene toxicity in *E. agilis* through Chl *a* fluorescence technique and the role of ROS in the overall toxicity response.

## Conclusions

Microalgae play a pivotal role in primary production in aquatic ecosystems, hence microalgal ingestion in polluted water bodies is a major route by which toxic chemicals can enter the food chain. The results of the present study confirm that anthracene exerts phytotoxic effects on *E. agilis* by disrupting growth, pigmentation and photosynthesis. Any severe reduction in these parameters will be followed by a loss of ecological competence and diminished survival of the entire *E. agilis* population, which could have a devastating impact on associated food chains.

Five principal conclusions derived from this study are:

1. The addition of anthracene resulted in a concentration-dependent reduction in cellular growth which appears to be highly related to a reduction in photosynthesis.
2. Anthracene had a pronounced negative effect on photosynthetic pigment content and a simultaneous reduction in all three photosynthetic pigments suggests that the major target of anthracene toxicity is the thylakoid compartment of chloroplasts. These results also indicate that in addition to causing a severe reduction in growth, anthracene exposure may reduce photosynthetic performance via the destruction of pigments responsible for harvesting available photons.
3. Toxicity of anthracene on Y(II), rETR_max_ and non-photochemical quenching parameters in microalgae taxa has for the first time been reported in the current study. The PSII quantum yield parameters collectively indicate the reduced efficiency of photochemical energy regulation, impairment of electron transport downstream from PSII, and an elevated reduction of the PQ pool imposed by anthracene exposure.
4. There was a significant increase in DCFH-DA fluorescence emission in anthracene exposed *E. agilis* cells, indicating a rise in the intracellular ROS levels. A probable source of generation of ROS would be an inhibition of the electron transport chain at PSII which would have transferred energy from triplet chlorophylls to oxygen, forming singlet oxygen species. A corresponding increase in Y(NO) with increasing anthracene also confirmed that the fraction of absorbed energy might have been used for the generation of free radicals (ROS) via an apparent catalytic transfer of electrons that occurred from the reduced PQ pool to O_2_.
5. Growth and Chl *a* content of *E. agilis* may serve as sensitive risk assessment parameters of anthracene toxicity in water management since EC_50_ values for both overlap with anthracene levels (8.3 mg L^−1^) permitted by the US Environmental Protection Agency (USEPA).

## Data Availability

Data can be obtained by contacting the corresponding author.

## References

[1] Gala WR, Giesy JP (1992). Photo–induced toxicity of anthracene to the green alga, *Selenastrum capricornutum*. Archives of Environmental Contamination and Toxicology.

[2] Chaudhry, G. R. Biological degradation and bioremediation of toxic chemicals. Dioscorides Press, Portland, OR, USA (1994).

[3] Behera BK (2018). Polycyclic Aromatic Hydrocarbons (PAHs) in inland aquatic ecosystems: Perils and remedies through biosensors and bioremediation. Environmental Pollution.

[4] Andersson TA (2005). Solubility of Acenaphthene, Anthracene, and Pyrene in Water At 50 °C to 300 °C. Journal of Chemical & Engineering Data.

[5] Krylov SN (1997). Mechanistic quantitative structure–activity relationship model for the photoinduced toxicity of polycyclic aromatic hydrocarbons: I Physical model based on chemical kinetics in a two–compartment system. Environmental Toxicology and Chemistry.

[6] Mallakin A (2002). Sites of toxicity of specific photooxidation products of anthracene to higher plants: inhibition of photosynthetic activity and electron transport in *Lemna gibba* L G–3 (duckweed). Environmental Toxicology.

[7] USEPA (U.S. Environmental Protection Agency), National recommended water quality criteria for priority pollutants. Office of Water, USEPA, Washington, DC (2009).

[8] Canadian Council of Ministers of the Environment, Canadian water quality guidelines for the protection of aquatic life: Polycyclic aromatic hydrocarbons (PAHs). In: Canadian environmental quality guidelines, Canadian Council of Ministers of the Environment, Winnipeg (1999).

[9] Wang XS (1996). Modeling the bioconcentration of hydrophonic organic chemicals in aquatic organisms. Chemosphere.

[10] Marwood AC (1999). Intact and photomodified polycyclic aromatic hydrocarbons inhibit photo– synthesis in natural assemblages of Lake Erie phytoplankton exposed to solar radiation. Ecotoxicology and Environmental Safety.

[11] Grote M (2005). Modeling photoinduced algal toxicity of polycyclic aromatic hydrocarbons. Environmental Science & Technology.

[12] Bi X (2016). Effect of anthracene (ANT) on growth, microcystin (MC) production and expression of MC synthetase (mcy) genes in *Microcystis aeruginosa*. Water, Air, & Soil Pollution.

[13] Brack W (2003). Identification of toxic products of anthracene photomodification in simulated sunlight. Environmental Toxicology and Chemistry.

[14] Erzinger, G. S. & Häder, D.-P. 4-Regulations, political and societal aspects, toxicity limits, Bioassays. Elsevier, 51–67 (2018).

[15] Aronsson KA, Ekelund NGA (2004). Biological eff ects of wood ash application to forest and aquatic ecosystems. Journal of Environmental Quality.

[16] Engel F (2015). Comparative toxicity of physiological and biochemical parameters in *Euglena gracilis* to short-term exposure to potassium sorbate. Ecotoxicology.

[17] Aronsson KA, Ekelund NGA (2005). Effects on motile factors and cell growth of *Euglena gracilis* after exposure to wood ash solution; assessment of toxicity, nutrient availability and pH–dependency. Water, Air, & Soil Pollution.

[18] De Filippis LF (1981). The effect of sublethal concentrations of zinc, cadmium and mercury on *Euglena*. Archives of Microbiology.

[19] Navarro L (1997). Comparison of physiological changes in *Euglena gracilis* during exposure to heavy metals of heterotrophic and autotrophic cells. Comparative Biochemistry and Physiology Part C: Pharmacology, Toxicology and Endocrinology.

[20] Dos Santos FVV (2007). Gene expression patterns in *Euglena gracilis*: insights into the cellular response to environmental stress. Gene.

[21] Ahmed H, Häder D-P (2010). A fast algal bioassay for assessment of copper toxicity in water using *Euglena gracilis*. Journal of Applied Phycology.

[22] Pettersson M, Ekelund NGA (2006). Effects of the herbicides Roundup and Avans on *Euglena gracilis*. Archives of Environmental Contamination and Toxicology.

[23] Li M (2009). Genotoxicity of organic pollutants in source of drinking on microalga *Euglena gracilis*. Ecotoxicology.

[24] Nestler H (2012). Multiple-endpoint assay provides a detailed mechanistic view of responses to herbicide exposure in *Chlamydomonas reinhardtii*. Aquatic toxicology.

[25] Sebastian C (1994). Effects of solar and artificial ultraviolet-radiation on pigment composition and photosynthesis in three Prorocentrum strains. Journal of Experimental Marine Biology and Ecology.

[26] Checcucci A (1976). Action spectra for photo–accumulation of green and colorless *Euglena*: evidence for identification of receptor pigments. Photochemistry and Photobiology.

[27] Jeffrey SW, Humphrey GF (1975). New spectrophotometric equations for determining chlorophylls a, b, c1 and c2 in higher plants, algae and natural phytoplankton. Plant Physiology and Biochemistry.

[28] Jassby AD, Platt T (1976). Mathematical formulation of the relationship between photosynthesis and light for phytoplankton. Limnology and oceanography.

[29] Maxwell K, Johnson GN (2000). Chlorophyll fluorescence—a practical guide. Journal of experimental botany.

[30] Rastogi RP (2010). Detection of reactive oxygen species (ROS) by the oxidant–sensing probe 2′,7′–dichlorodihydrofluorescein diacetate in the cyanobacterium *Anabaena variabilis* PCC 7937. Biochemical and Biophysical Research Communications.

[31] Okumura Y (2001). Influence of organic solvents on the growth of marine microalgae. Archives of Environmental Contamination and Toxicology.

[32] Aksmann A, Tukaj Z (2004). The effect of anthracene and phenanthrene on the growth, photosynthesis and SOD activity of green alga *Scenedesmus armatus* depend on the PAR irradiance and CO_2_ level. Archives of Environmental Contamination and Toxicology.

[33] Gala WR, Giesy JP (1993). Using the carotenoid biosynthesis inhibiting herbicide, Fluridone, to investigate the ability of carotenoid pigments to protect algae from the photoinduced toxicity of anthracene. Aquatic Toxicology.

[34] Marwood CA (2001). Chlorophyll fluorescence as a bioindicator of effects on growth in aquatic macrophytes from mixtures of polycyclic aromatic hydrocarbons. Environmental Toxicology and Chemistry.

[35] Pokora W, Tukaj Z (2010). The combined effect of anthracene and cadmium on photosynthetic activity of three *Desmodesmus* (Chlorophyta) species. Ecotoxicology and Environmental Safety.

[36] Sikkema J (1992). Effects of the membrane action of Tetralin on the functional and structural properties of artificial and bacterial membranes. Journal of Bacteriology.

[37] Hager A, Stransky H (1970). Das Carotenoidmuster und die Verbreitung des lichtinduzierten Xanthophyllcyclus in verschiedenen Algenklassen. Archives of Microbiology.

[38] Aksmann A, Tukaj Z (2008). Intact anthracene inhibits photosynthesis in algal cells: a fluorescence induction study on *Chlamydomonas reinhardtii* cw92 strain. Chemosphere.

[39] Duxbury CL (1997). Effects of simulated solar radiation on the bioaccumulation of polycyclic aromatic hydrocarbons by the duckweed *Lemna gibba*. Environmental Toxicology and Chemistry.

[40] Garcia DC (2011). Physiological responses of *Euglena gracilis* to copper stress. Quimica Nova.

[41] Middleton EM, Teramura AH (1993). The role of flavonol glycosides and carotenoids in protecting soybean from UV–B damage. Plant Physiology.

[42] Hutchinson TC (1980). The correlation of the toxicity to algae of hydrocarbons and halogenated hydrocarbons with their physical–chemical properties. Environmental Science and Pollution Research.

[43] Sayyad-Amin P (2016). Changes in photosynthetic pigments and chlorophyll-a fluorescence attributes of sweet-forage and grain sorghum cultivars under salt stress. Journal of Biological Physics.

[44] Huang X-D (1997). Mechanisms of photoinduced toxicity of photomodified anthracene to plants: inhibition of photosynthesis in the aquatic higher plant *Lemna gibba* (duckweed). Environmental Toxicology and Chemistry.

[45] Rocchetta I, Küpper H (2009). Chromium– and copper–induced inhibition of photosynthesis in *Euglena gracilis* analysed on the single–cell level by fluorescence kinetic microscopy. New Phytologist.

[46] Briantais JM (1979). A quantitative study of the slow decline of chlorophyll a fluorescence in isolated chloroplasts. Biochimica et Biophysica Acta.

[47] González–Moreno S (1997). Multiple effects of salinity on photosynthesis of the protist *Euglena gracilis*. Physiologia Plantarum.

[48] Krieger–Liszkay A (2004). Singlet oxygen production in photosynthesis. Journal of Experimental Botany.

[49] Apel K, Hirt H (2004). Reactive oxygen species: metabolism, oxidative stress, and signal transduction. Annual Review of Plant Biology.

[50] Kottuparambil S (2012). UV–B affects photosynthesis, ROS production and motility of the freshwater flagellate. Euglena agilis Carter, Aquatic Toxicology.

[51] Pätsikkä E (1998). Increase in quantum yield of photoinhibition contributes to copper toxicity *in vivo*. Plant Physiology.

[52] Babu TS (2001). Synergistic effects of a photooxidized polycyclic aromatic hydrocarbon and copper on photosynthesis and plant growth: evidence that *in vivo* formation of reactive oxygen species is a mechanism of copper toxicity. Environmental Toxicology and Chemistry.

[53] Pavelescu, L. On reactive oxygen species measurement in living systems. *Journal of medicine and life* **8**(Spec Issue), 38 (2015). PMC4564046 26361509

[54] Wang J (2011). Generation of reactive oxygen species in cyanobacteria and green algae induced by allelochemicals of submerged macrophytes. Chemosphere.

[55] He YY, Häder DP (2002). Reactive oxygen species and UV-B: effect on cyanobacteria. Photochemical & Photobiological Sciences.

[56] Sánchez-Thomas R (2016). Accumulation of zinc protects against cadmium stress in photosynthetic *Euglena gracilis*. Environmental and Experimental Botany.

[57] Garcia-Garcia JD (2016). Bio-recovery of non-essential heavy metals by intra-and extracellular mechanisms in free-living microorganisms. Biotechnology advances.

[58] Rocchetta I (2006). Effect of chromium on the fatty acid composition of two strains of *Euglena gracilis*. Environmental pollution.

[59] Klughammer C, Schreiber U (2008). Saturation pulse method for assessment of energy conversion in PSI. PAM Application Notes.

[60] Lu GN (2008). Estimation of n-octanol/water partition coefficients of polycyclic aromatic hydrocarbons by quantum chemical descriptors. Open Chemistry.

[61] OECD, O. Guidance Document on Acute Oral Toxicity Testing. (2000).

[62] Patel, J. G., Kumar, J. I. N., Kumar, R. N. & Khan, S. R. Biodegradation Capability and Enzymatic Variation of Potentially Hazardous Polycyclic Aromatic Hydrocarbons—Anthracene and Pyrene by. Polycyclic Aromatic Compounds **36**(1), 72–87 (2015).

[63] Cody T.E., Radike M.J., Warshawsky D. (1984). The phototoxicity of benzo[a]pyrene in the green alga Selenastrum capricornutum. Environmental Research.

[64] Yan, X. *et al*. Study on the toxicological response of Chlo rella to anthracene under different nutrient conditions. Journal of Wuhan University (Natural Science Edition) **45**(6), 845–848 (1999).

[65] Djomo J.E., Dauta A., Ferrier V., Narbonne J.F., Monkiedje A., Njine T., Garrigues P. (2004). Toxic effects of some major polyaromatic hydrocarbons found in crude oil and aquatic sediments on Scenedesmus subspicatus. Water Research.

[66] Tukaj, Z. & Pokora, W. Individual and combined effect of anthracene, cadmium, and chloridazone on growth and activity of SOD izoformes in three Scenedesmus species. *Ecotoxicology and Environmental Safety* **65**(3), 323–331 (2006). 10.1016/j.ecoenv.2005.12.001 16464497

[67] Baścik-Remisiewicz Agnieszka, Aksmann Anna, Żak Adam, Kowalska Maja, Tukaj Zbigniew (2010). Toxicity of Cadmium, Anthracene, and Their Mixture to Desmodesmus subspicatus Estimated by Algal Growth-Inhibition ISO Standard Test. Archives of Environmental Contamination and Toxicology.

[68] Bi, X. *et al*. Effect of Anthracene (ANT) on growth, microcystin (MC) production and expression of MC synthetase (mcy) genes in Microcystis aeruginosa. 10.1038/s41598-019-51451-y Water, Air, & Soil Pollution **227**(8) (2016).

